# Multi-Omics Analysis to Examine Gene Expression and Metabolites From Multisite Adipose-Derived Mesenchymal Stem Cells

**DOI:** 10.3389/fgene.2021.627347

**Published:** 2021-02-18

**Authors:** Chuanxi Yang, Jing Zhang, Tingting Wu, Kun Zhao, Xiaoguang Wu, Jing Shi, Wei Sun, Xiangqing Kong

**Affiliations:** ^1^Department of Cardiology, Medical School of Southeast University, Nanjing, China; ^2^Department of Cardiology, The First Affiliated Hospital of Nanjing Medical University, Nanjing, China

**Keywords:** adipose-derived mesenchymal stem cells, gene expression, metabolites, RNA-seq, metabolomics

## Abstract

This study aimed at exploring the gene expression and metabolites among multisite adipose-derived mesenchymal stem cells (ASCs) and investigate the metabolic pathway using a multi-omics analysis. Subcutaneous adipose-derived mesenchymal stem cells (SASCs), perirenal adipose-derived mesenchymal stem cells (PASCs), and epididymal adipose-derived mesenchymal stem cells (EASCs) were isolated from Sprague Dawley rats. RNA and metabolites were extracted and sequenced using transcriptomics and metabolomics analyses, respectively. There were 720 differentially expressed genes (DEGs) in EASCs and 688 DEGs in PASCs compared with SASCs; there were 166 unique DEGs in EASCs, 134 unique DEGs in PASCs, and 554 common DEGs between EASCs and PASCs. Furthermore, there were 226 differential metabolites in EASCs, 255 differential metabolites in PASCs, 83 unique differential metabolites in EASCs, 112 unique differential metabolites in PASCs, and 143 common differential metabolites between EASCs and PASCs. The transcriptomics and metabolomics analyses identified four hub genes, one in EASCs and three in PASCs. There are functional differences among multisite ASCs that may be related to the hub genes *Atac2*, *Rrm1*, *Rrm2*, and *Gla*. The relevant signaling pathways are the Ras signaling pathway, HIF-1 signaling pathway, and the p53 signaling pathway. In conclusion, compared with SASCs, our multi-omics analysis identified that EASCs with higher *Acat2* expression may be more correlated to fat metabolism and insulin resistance, while PASCs with abnormal expression of *Rrm1/2* and *Gla* may be more correlated with some malignant tumors and cardiac-cerebral vascular disease.

## Introduction

Obesity, which poses a grave threat to humankind, has become one of the most important chronic diseases. By 2016, more than 1.9 billion adults over 18 years of age were overweight worldwide, of whom more than 650 million were obese (Chandramouli et al., 2019). Obesity has become an increasing concern because it is a major risk factor for diseases including cardiovascular disease, hypertension, dyslipidemia, diabetes, and cancer (Lopez et al., 2006; Renehan et al., 2008; Aune et al., 2016).

As early as 1956, Vague (1956) proposed that obesity as an important risk factor for cardiovascular disease is related to the distribution of adipose tissue in the human body, not just the increase of total adipose tissue. There are two types of adipose tissue in mammals: white adipose tissue (WAT), which is the main form of energy storage in the body, and brown adipose tissue (BAT). WAT is primarily composed of subcutaneous adipose tissue (SAT) and visceral adipose tissue (VAT). WAT can be enlarged by increasing adipocyte size (hypertrophy) or adipocyte number (hyperplasia); adipocyte hypertrophy is associated with adipose tissue dysfunction and inflammation, whereas hyperplasia is involved in the improvement of insulin sensitivity (Silva and Baptista, 2019). Previous studies have shown that the expansion of SAT is dominated by adipocyte hyperplasia, while VAT [including omental, mesenteric, and perirenal adipose tissue (PAT)] is primarily increased by adipocyte hypertrophy (Joe et al., 2009). Compared with SAT and VAT secretes higher levels of proinflammatory factors and lower levels of anti-inflammatory factors; therefore, it is easier to break down to increase plasma-free fatty acids (FFA). High concentrations of FFA can inhibit insulin signaling pathways in skeletal muscle and liver, induce insulin resistance, and increase the risk of diabetes and cardiovascular diseases (McFarlane et al., 2001; Sharma et al., 2001; Rocchini, 2002). Therefore, the accumulation of VAT is more closely associated with cardiovascular disease, insulin resistance, hypermetabolism, and cancer (Dobbelsteyn et al., 2001; Fox et al., 2007; Tran et al., 2008; Tchernof and Després, 2013; Silva and Baptista, 2019). In addition, compared with other VATs [such as epididymal adipose tissue (EAT)], PAT has some characteristics of BAT. In adults, PAT depot is one of the most frequent depots where BAT have been found (Svensson et al., 2014). Moreover, the expression of UCP1 in PAT is related to blood pressure (Li et al., 2015). Whereas, some investigators indicate that EAT maximum thickness is better associated with cardiovascular risk factors (Kawamoto et al., 2002; Nishina et al., 2003).

Adipose tissue is mainly composed of adipocytes (90%), nonadipocytes (preadipocytes, fibroblasts, endothelial cells, immune cells, inflammatory cells, etc.), connective tissue matrix, blood vessels, and nervous tissues (Ibrahim, 2010). Girousse et al. (2019) stated that adipocytes mainly originate from adipose-derived mesenchymal stem cells (ASCs), and Silva and Baptista (2019) demonstrated the different pathophysiologic properties of ASCs in different adipose deposits. A widely different function among Subcutaneous adipose-derived mesenchymal stem cells (SASCs), epididymal adipose-derived mesenchymal stem cells (EASCs), and perirenal adipose-derived mesenchymal stem cells (PASCs) were illustrated including a higher potential of lipogenesis in EASCs and PASCs (Jeffery et al., 2015, 2016). However, the contributions of different original ASCs in different adipose deposits are still not clear. We designed this study to investigate the functional differences among different original ASCs by multi-omics analysis.

## Results

### Functional Annotation of DEGs Among SASCs, PASCs, and EASCs

To explore the functional differences of SASCs, PASCs, and EASCs, 720 differentially expressed genes (DEGs) between EASCs and SASCs (FC >2.0, adjusted *P*-value (FDR) <0.05, 497 downregulated, and 223 upregulated), and 688 DEGs between PASCs and SASCs (FC >2.0, FDR 0.05, 468 downregulated, and 220 upregulated) were found by RNA-Seq. Using Metacore system, the top 10 different enrichments in pathway maps, GO processes, and process networks of DEGs in EASCs and PASCs are shown in Supplementary Figures 1A,B. In addition, the top scored network analyses of DEGs in EASCs were involved in regulation of smooth muscle cell proliferation (25.6%), regulation of cell population proliferation (48.8%), digestive system development (20.9%), positive regulation of smooth muscle cell proliferation (18.6%), and positive regulation of developmental process (44.2%). Whereas, DEGs in PASCs were most involved in negative regulation of GTPase activity (12.0%), negative regulation of hydrolase activity (24.0%), oncostatin-M-mediated signaling pathway (8.0%), leukemia inhibitory factor signaling pathway (8.0%), and regulation of hydrolase activity (34.0%) (Supplementary Figures 1C,D).

#### Upregulated DEGs

**FIGURE 1:**
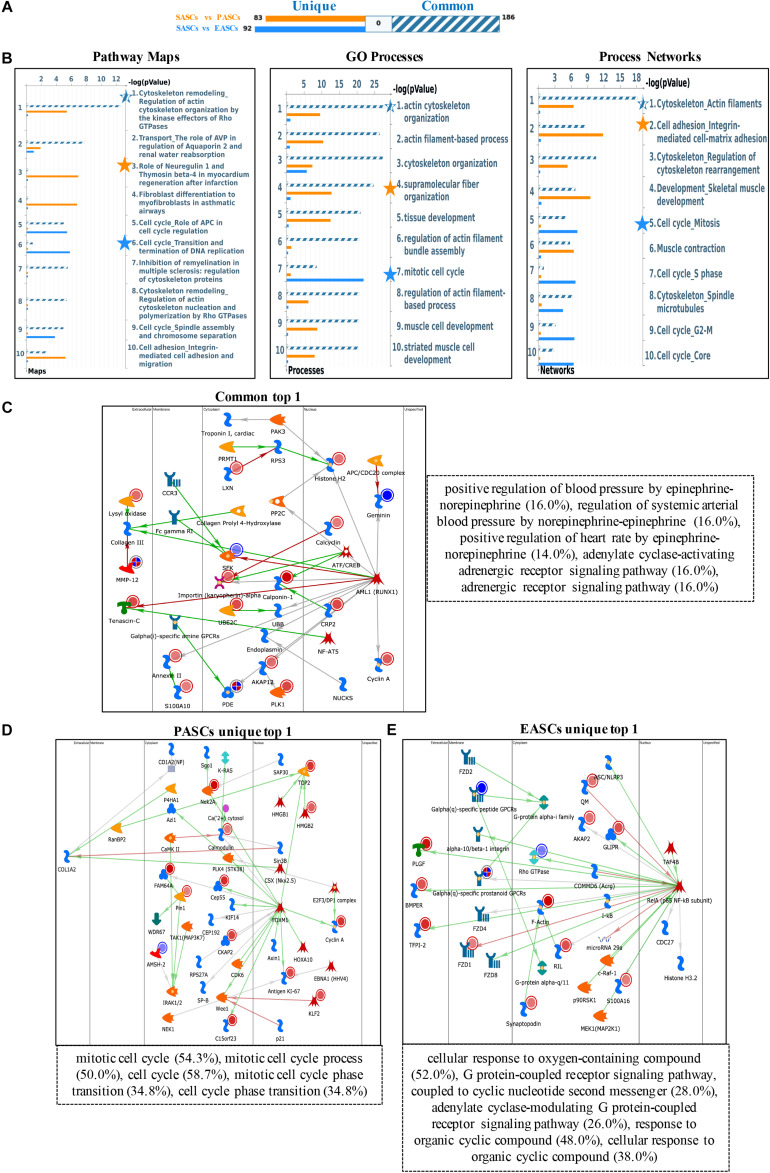
Functional annotation of upregulated DEGs among SASCs, PASCs, and EASCs. **(A)** The gene content is aligned between EASCs vs. SASCs with PASCs vs. SASCs. The intersection set of experiments is defined as “common” and marked as a blue/white striped bar. The unique genes for the experiments are marked as colored bars. **(B)** Top 10 pathway map analysis (left panel), Gene Ontology (GO) process analysis (middle panel), and process networks (right panel) analysis sorting as differentially affected for different sets of DEGs. Star filling with different colors represent the significant enrichment. **(C)** Top scored network analysis of common upregulated DEGs. **(D)** Top scored network analysis of unique upregulated DEGs in PASCs. **(E)** Top scored network analysis of unique upregulated DEGs in EASCs.

Specifically, the upregulated DEGs show different enrichment in pathway maps: regulation of actin cytoskeleton organization by the kinase effectors of Rho GTPases in common DEGs, role of neuregulin 1 and thymosin beta-4 in myocardium regeneration after infarction in PASCs, and transition and termination of DNA replication in EASCs. Gene Ontology (GO) process enrichment analyses show the common DEGs are most relevant to actin cytoskeleton organization, the PASCs are strongly associated with supramolecular fiber organization, and the EASCs are most enriched in the mitotic cell cycle. In addition, actin filaments in common DEGs, integrin-mediated cell-matrix adhesion in PASCs, and mitosis in EASCs were seen in process networks (Figures 1A,B).

In particular, the top scored network analyses of common upregulated DEGs were found to be involved in positive regulation of blood pressure by epinephrine-norepinephrine (16.0%), regulation of systemic arterial blood pressure by norepinephrine-epinephrine (16.0%), positive regulation of heart rate by epinephrine-norepinephrine (14.0%), adenylate cyclase-activating adrenergic receptor signaling pathway (16.0%), and adrenergic receptor signaling pathway (16.0%) (Figure 1C). The unique upregulated DEGs in PASCs were most participated in the mitotic cell cycle (54.3%), mitotic cell cycle process (50.0%), cell cycle (58.7%), mitotic cell cycle phase transition (34.8%), and cell cycle phase transition (34.8%) (Figure 1D). The unique upregulated DEGs in EASCs showed the top scored network is a cellular response to oxygen-containing compound (52.0%), G protein-coupled receptor signaling pathway, coupled to cyclic nucleotide second messenger (28.0%), adenylate cyclase-modulating G protein-coupled receptor signaling pathway (26.0%), response to organic cyclic compound (48.0%), and cellular response to organic cyclic compound (38.0%) (Figure 1E).

#### Downregulated DEGs

**FIGURE 2:**
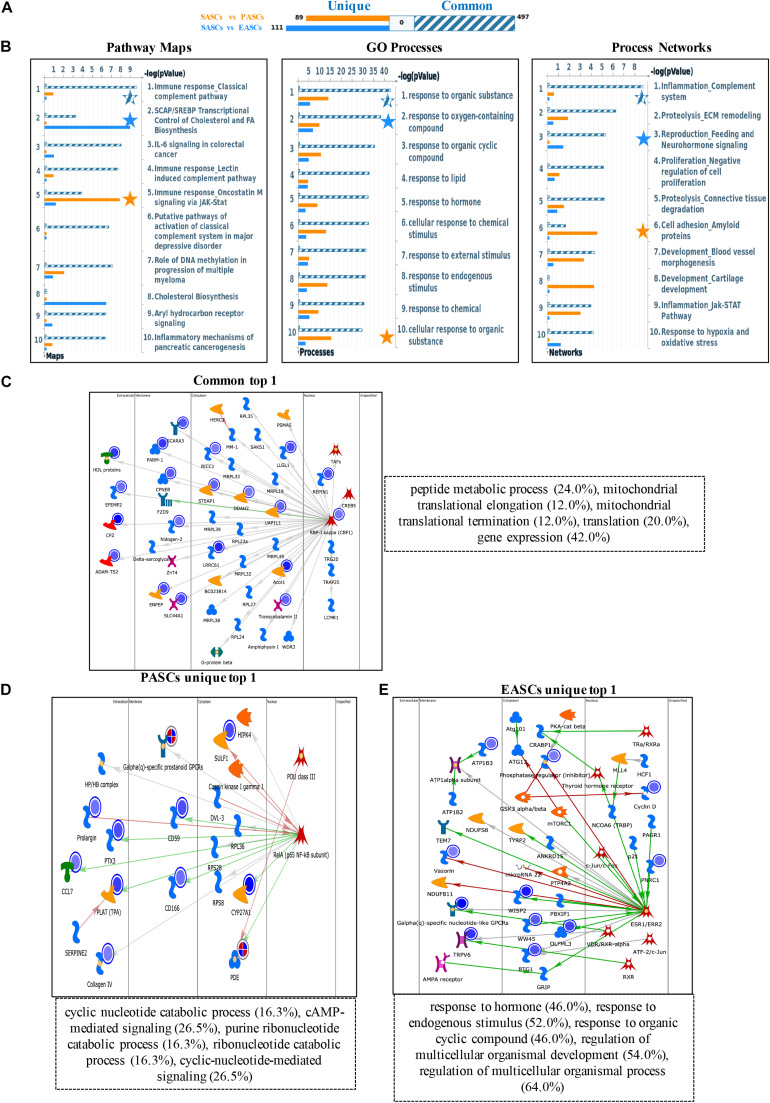
Functional annotation of downregulated DEGs among SASCs, EASCs, and PASCs. **(A)** The gene content is aligned between EASCs vs. SASCs with PASCs vs. SASCs. The intersection set of experiments is defined as “common” and marked as a blue/white striped bar. The unique genes for the experiments are marked as colored bars. **(B)** Top 10 pathway map analysis (left panel), Gene Ontology (GO) process analysis (middle panel), and process networks (right panel) analysis sorting as differentially affected for different sets of DEGs. Star filling with different color represent the significant enrichment. **(C)** Top scored networks analysis of common downregulated DEGs. **(D)** Top scored networks analysis of unique downregulated DEGs in PASCs. **(E)** Top scored networks analysis of unique downregulated DEGs in EASCs.

To further explore the different functions among the ASCs derived from three adipose deposits, we performed multilevel analyses including pathway maps, GO processes, and process networks with downregulated DEGs. First, common downregulated DEGs were enriched in the classical complement pathway about pathway maps, response to organic substance about GO processes, and complement system about process networks. Second, the unique downregulated DEGs of PASCs were involved in SCAP/SREBP transcriptional control of cholesterol and FA biosynthesis about pathway maps, response to oxygen-containing compound about GO processes, and feeding and neurohormone signaling about process networks. Third, the unique downregulated DEGs of EASCs were most relevant to oncostatin M signaling *via* Jak-Stat about pathway maps, cellular response to organic substance about GO processes, and amyloid proteins about process networks (Figures 2A,B).

Similarly, the top scored network analyses of common downregulated DEGs were found to be most associated with peptide metabolic process (24.0%), mitochondrial translational elongation (12.0%), mitochondrial translational termination (12.0%), translation (20.0%), and gene expression (42.0%) (Figure 2C). The unique downregulated DEGs in PASCs were involved in cyclic nucleotide catabolic process (16.3%), cAMP-mediated signaling (26.5%), purine ribonucleotide catabolic process (16.3%), ribonucleotide catabolic process (16.3%), and cyclic-nucleotide-mediated signaling (26.5%) (Figure 2D). Additionally, the unique downregulated DEGs in EASCs showed the top scored network is in response to hormone (46.0%), response to the endogenous stimulus (52.0%), response to organic cyclic compound (46.0%), regulation of multicellular organismal development (54.0%), and regulation of multicellular organismal process (64.0%) (Figure 2E).

### Analysis of Metabolic Pathways in SASCs, EASCs, and PASCs

To reduce systematic and technical bias, metabolomics data were normalized and the metabolic data were normally distributed (Supplementary Figure 2). Principal component analysis (PCA) suggested that SASCs vs. PASCs and SASCs vs. EASCs could be distinguished, allowing for the determination of differentiated metabolites (Supplementary Figure 2).

**FIGURE 3:**
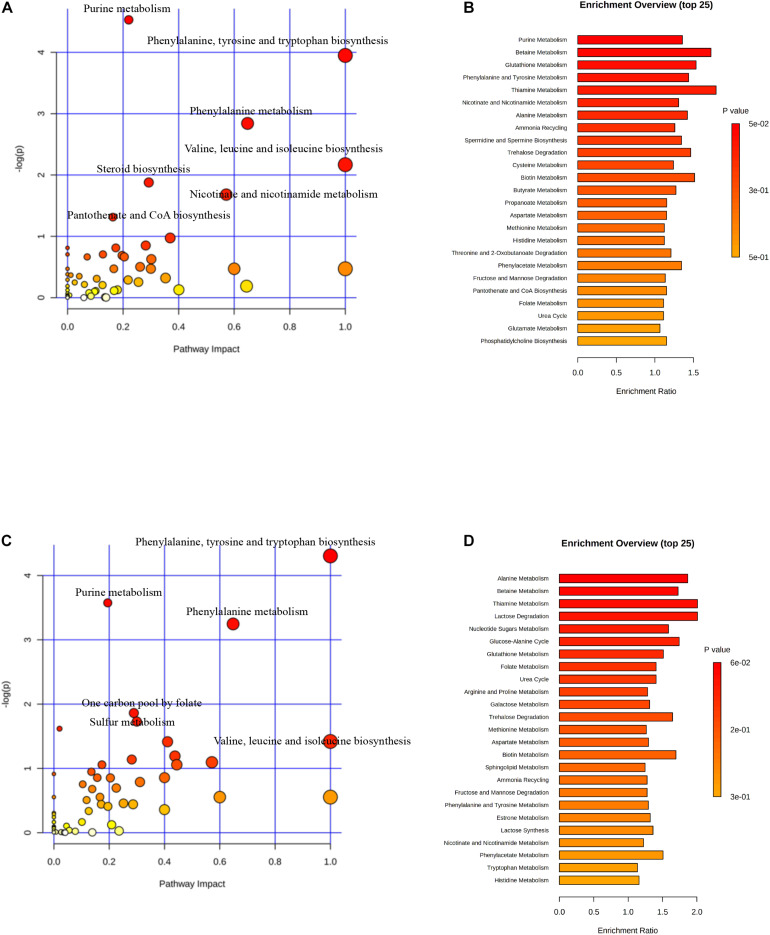
Analysis of metabolic pathways in SASCs, EASCs, and PASCs. **(A)** Analysis of metabolic pathways of differential metabolites between SASCs and PASCs. The color depends on the *P*-value, and the size of the circle represents the degree of correlation. **(B)** Top 25 enrichment of differential metabolites between SASCs and PASCs, and the *P*-value represents the enrichment level, the darker the orange, the greater the *P*-value; the length of the bar represents the amounts of metabolites. **(C)** Analysis of metabolic pathways of differential metabolites between SASCs and EASCs. **(D)** Top twenty-five enrichment of differential metabolites between SASCs and EASCs.

Two hundred and fifty-five differential metabolites in PASCs were found compared with SASCs (FC >2.0, FDR <0.05). MetaboAnalysis 3.0 was used to analyze the metabolic pathways of significantly different metabolites. The results showed that the differential metabolites were primarily involved in seven metabolic pathways (Figures 3A,B). Similarly, 226 (FC >2.0, FDR <0.05) differential metabolites were identified in EASCs, and the differential metabolites were primarily involved in six major metabolic pathways (Figures 3C,D). There were four common metabolic pathways between the two comparisons.

### Analysis of Unique Differential Metabolites Between EASCs and PASCs

**FIGURE 4:**
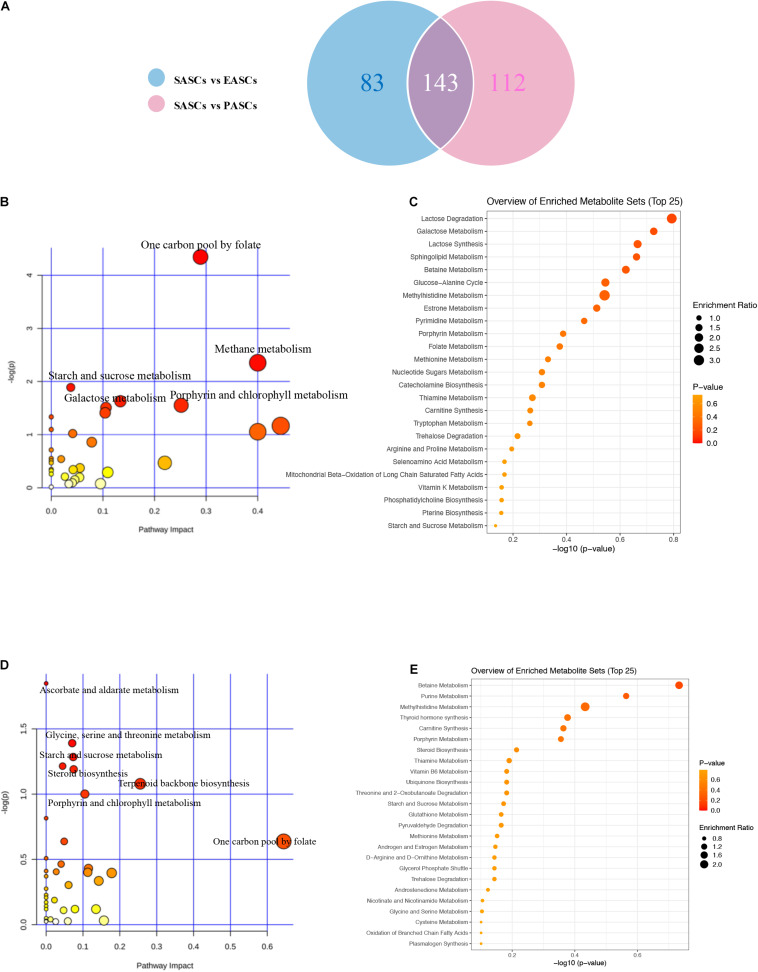
Analysis of unique differential metabolites in SASCs, EASCs, and PASCs. **(A)** A Wayne chart of differential metabolites between SASCs vs. EASCs with SASCs vs. PASCs. **(B)** Analysis of metabolic pathways of unique differential metabolites between SASCs and EASCs. **(C)** Overview of the enrichment of unique differential metabolites between SASCs and EASCs. **(D)** Analysis of metabolic pathways of unique differential metabolites between SASCs and PASCs. **(E)** Overview of the enrichment of unique differential metabolites between SASCs and PASCs.

To further explore the differences of differential metabolites of ASCs among the three adipose deposits, we analyzed the differential metabolites of SASCs vs. EASCs and SASCs vs. PASCs and found that compared with SASCs, there were 143 common differential metabolites between EASCs and PASCs (FC >2.0, FDR <0.05) (Supplementary Table 1), 83 unique differential metabolites in EASCs (FC >2.0, FDR <0.05) (Supplementary Table 2), and 112 unique differential metabolites in PASCs (FC >2.0, FDR <0.05) (Figure 4A and Supplementary Table 3). Pathway analysis revealed that the unique differential metabolites in EASCs involved one carbon pool by folate, methane metabolism, starch and sucrose metabolism, galactose metabolism, porphyrin, and chlorophyll metabolism (Figures 4B,C), whereas the unique metabolites in PASCs mainly involved six metabolic pathways, including ascorbate and aldarate metabolism; glycine, serine, and threonine metabolism; starch and sucrose metabolism; steroid biosynthesis; terpenoid backbone biosynthesis; porphyrin and chlorophyll metabolism; and one carbon pool by folate (Figures 4D,E).

### Integrative Analysis of Multi-Omics Data

**FIGURE 5:**
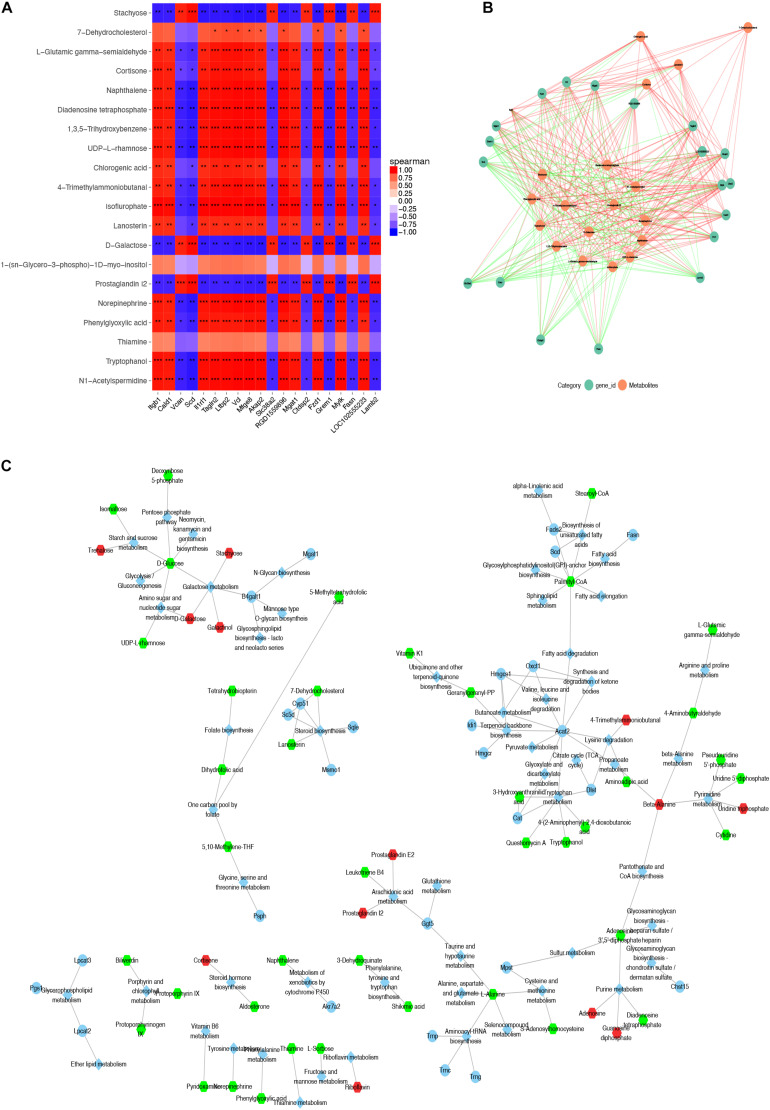
Combined analysis of DEGs and expression of differential metabolites in SASCs and EASCs. **(A)** A heat map of the top 20 DEGs and differential metabolites in SASCs and EASCs; the horizontal axis represents DEGs, and the vertical represents regulated metabolites. In the figure, red indicates a positive correlation, and blue indicates a negative correlation. The deeper the color, the greater the correlation. **(B)** The PPI network of the DEGs and differential metabolites. The red line indicates a positive correlation, the green line indicates a negative correlation, and the thickness of the line represents the correlation coefficient. **(C)** KGML network analysis of DEGs and differential metabolites in SASCs and EASCs. The circles represent genes, hexagons represent metabolites, and diamonds represent pathways. Red indicates upregulated genes or metabolites, and green indicates downregulated genes or metabolites. ***P < 0.001, correlation; **P < 0.01, correlation; *P < 0.05, correlation.

**FIGURE 6:**
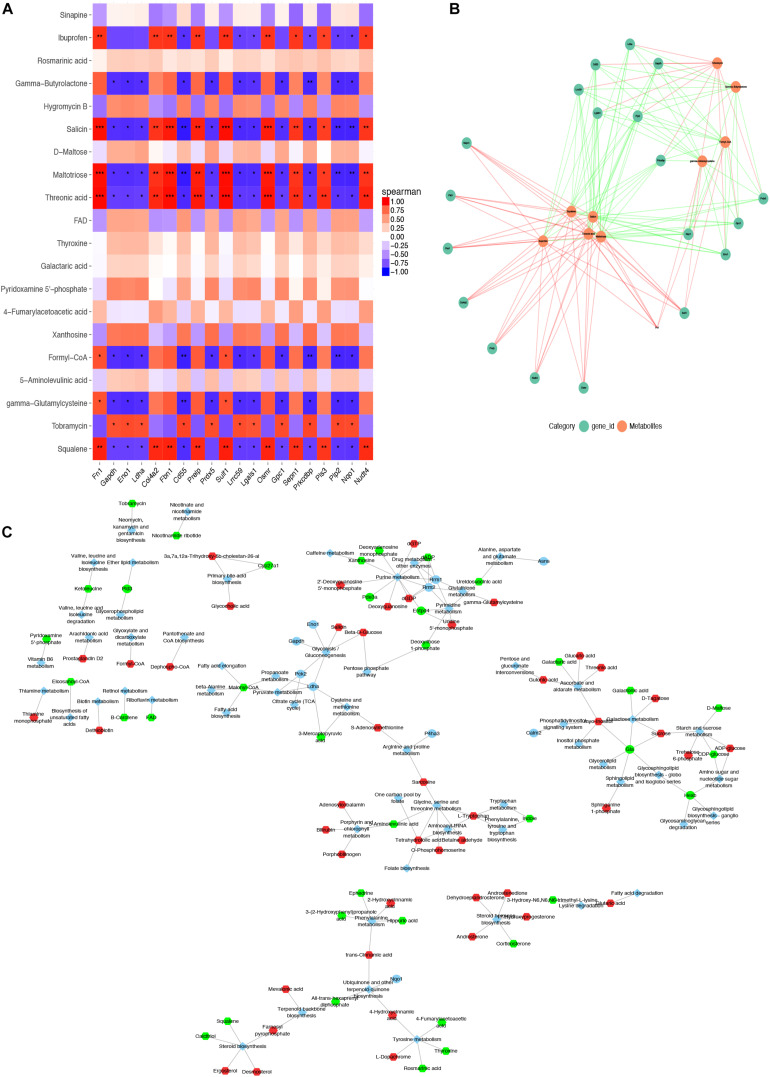
Combined analysis of DEGs and differential metabolites expression in SASCs and PASCs. **(A)** The heat map of the top 20 DEGs and differential metabolites in SASCs and PASCs. **(B)** The PPI network of the DEGs and differential metabolites. **(C)** KGML network analysis of DEGs and differential metabolites. ***P < 0.001, correlation; **P < 0.01, correlation; *P < 0.05, correlation.

We analyzed the association between DEGs and differential metabolites among three ASC adipose deposits. In EASCs, the heat map and association analysis network diagram show the top 20 DEGs and differential metabolites and the correlation between DEGs and metabolites by Pearson correlation analysis with *P*-value <0.05 (Figures 5A,B), including 18 differential metabolites and 19 DEGs. The results showed that the *acetyl coenzyme A acetyltransferase 2* (*Acat2*) gene (the top 10 nodes ranked by MCC and degree ≥6, Supplementary Figure 3 and Supplementary Table 4) was associated with nine metabolites in 10 metabolic pathways, including seven downregulated and two upregulated metabolites in the Kyoto Encyclopedia of Genes and Genomes (KEGG) Markup Language (KGML) network analysis (Figure 5C). Similarly, the correlation analysis between DEGs and differential metabolites in SASCs and PASCs showed that 20 DEGs were closely related to 9 differential metabolites. KGML network analysis showed that the *Rrm1/Rrm2* gene (the top 10 nodes ranked by MCC and degree ≥6, Supplementary Figure 3 and Supplementary Table 5) was closely related to 11 metabolites in six metabolic pathways, including five downregulated and six upregulated metabolites; the *Galactosidase alpha* (*Gla*) gene (the top 10 nodes ranked by MCC and degree ≥6, Supplementary Figure 3 and Supplementary Table 5) was closely related to four metabolites in six metabolic pathways, including one downregulated and three upregulated metabolites (Figures 6A–C).

### Analysis of Common DEGs and Metabolites Among SASCs, EASCs, and PASCs

**FIGURE 7:**
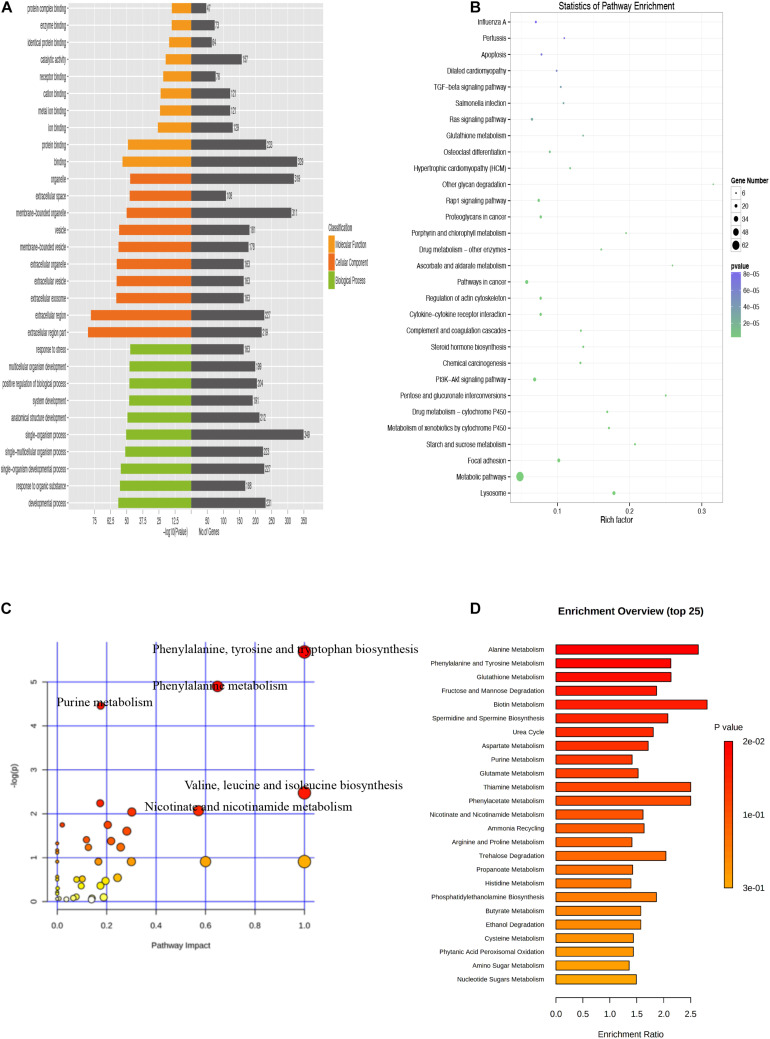
Analysis of common DEGs and metabolites among SASCs, EASCs, and PASCs. **(A)** Enriched GO terms of common DEGs among SASCs, EASCs, and PASCs. **(B)** Enriched KEGG pathways of common DEGs. **(C)** Analysis of metabolic pathways of common differential metabolites among SASCs, EASCs, and PASCs. **(D)** Top 25 enrichment of common differential metabolites.

**FIGURE 8:**
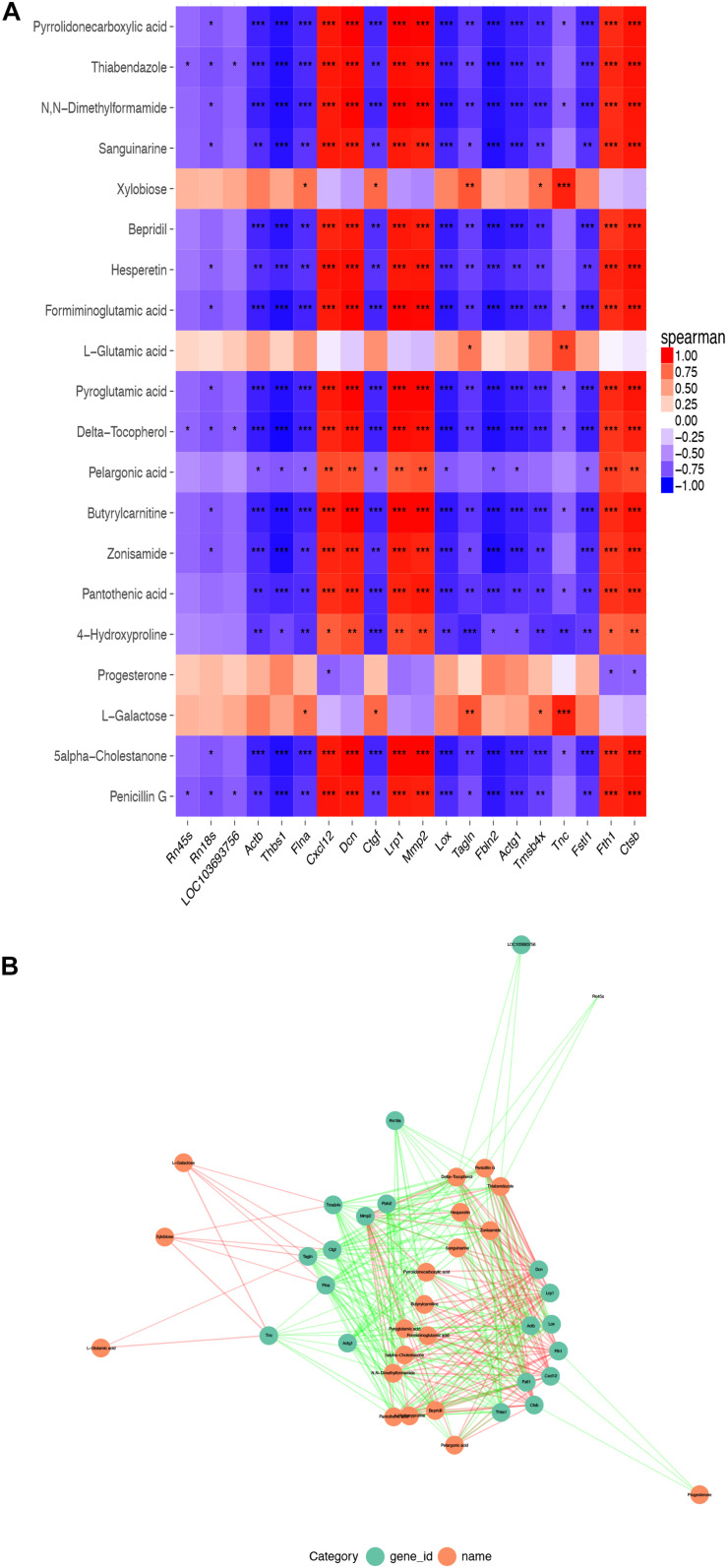
Correlation heat map and correlation network map of common DEGs and differential metabolites. **(A**) A heat map of the top 20 common DEGs and differential metabolites among SASCs, EASCs, and PASCs. **(B)** The PPI network of common DEGs and differential metabolites. Notes: ***P < 0.001, correlation*;* **P < 0.01, correlation; *P < 0.05, correlation.

There were 554 common trend DEGs (FC >2.0, FDR <0.05) (Supplementary Table 6) and 143 common differential metabolites (FC >2.0, FDR <0.05) (Supplementary Table 1) among SASCs, EASCs, and PASCs. GO analysis of common DEGs showed the top 10 significantly enriched BPs, CCs, and MFs (Figure 7A). The results of the KEGG analysis showed that common DEGs participated in several information processing pathways including apoptosis, the TGF-beta signaling pathway, Rap1 signaling pathway, cytokine–cytokine receptor interaction, regulation of actin cytoskeleton, PI3K-Akt signaling pathway, focal adhesion, and lysosome (Figure 7B). Common differential metabolites were primarily involved in five metabolic pathways, including phenylalanine, tyrosine, and tryptophan biosynthesis; phenylalanine metabolism; purine metabolism; valine, leucine, and isoleucine biosynthesis; and nicotinate and nicotinamide metabolism (Figures 7C,D). Figures 8A,B shows the results of the correlation heat map and correlation network map of common DEGs and differential metabolites.

## Discussion

Adipose tissue, which is divided into BAT and WAT, including SAT and VAT, is the main metabolic store in the human body. Compared with VAT, the enlargement of the SAT can be beneficial to patients by improving insulin sensitivity and reducing the risk of type II diabetes (Hoffstedt et al., 2010; Arner et al., 2011; Dalmas et al., 2015). Conversely, excessive accumulation of VAT increases the risk of diseases such as type II diabetes and cardiovascular disease.

According to previous studies, VAT secretes higher levels of proinflammatory factors and lower levels of anti-inflammatory factors, decomposes FFA, and increases plasma more easily than SAT. As reported, high concentrations of FFA can inhibit insulin signaling pathways in skeletal muscle and liver, induce insulin resistance, and eventually increase the risk of diabetes and cardiovascular diseases. In addition, blood pressure can be elevated by activation of the renin-angiotensin-aldosterone system, the sympathetic nervous system, and mechanisms related to insulin resistance (McFarlane et al., 2001; Sharma et al., 2001; Rocchini, 2002). Therefore, measuring waist circumference to quantify VAT can be used as an independent risk factor for type II diabetes, cardiovascular disease, and cancer (Dobbelsteyn et al., 2001; Tchernof and Després, 2013).

In this study, SASCs, EASCs, and PASCs were extracted from three different adipose deposits in rats, and transcriptomics and metabolomics analyses were used to reveal that there were 720 DEGs in EASCs and 688 DEGs in PASCs compared with SASCs. In addition, there were 166 unique DEGs in EASCs (Supplementary Table 7), 134 unique DEGs in PASCs (Supplementary Table 8), and 554 common DEGs between EASCs and PASCs. Furthermore, there were 226 differential metabolites in EASCs, 255 differential metabolites in PASCs, 83 unique differential metabolites in EASCs, 112 unique differential metabolites in PASCs, and 143 common differential metabolites between EASCs and PASCs.

Collectively, we found that immune response and cytoskeleton remodeling in pathway maps, response to organic substance and response to organic cyclic compound in GO processed, and cytoskeleton and cell adhesion in process networks are common in EASCs and PASCs. Interestingly, cytoskeleton remodeling was the most enrichment of upregulated DEGs both in EASCs and PASCs, while immune response was the most enrichment of downregulated DEGs both in EASCs and PASCs. Also, KEGG analysis (Supplementary Figure 4) showed ASCs derived from different deposits of VAT were both involved in cytokine–cytokine receptor interaction, Rap1 signaling pathway, regulation of actin cytoskeleton, PI3K-Akt signaling pathway, lysosome, and focal adhesion. The PI3K-Akt signaling pathway has been reported to primarily regulate the transcription, translation, proliferation, growth, survival, and other basic functions of extracellular signals. These new findings proved that the functions of ASCs derived from different deposits of VAT were similar, while different functions of immune response and extracellular matrix remodeling vary compared with the ASCs derived from SAT. As reported previously, the adipose tissue from subcutaneous and visceral showed a significant difference in immune response in obesity and other metabolic diseases (Sharma et al., 2001; Klimcakova et al., 2011). Compared with our studies, these differences may be most relevant to the different ASCs from SAT and VAT. Whereas, further studies on this point are required for confirmation. Furthermore, the function of extracellular matrix remodeling was reported as a critical microenvironment in different sites of adipose tissue (Mori et al., 2014), and, by standard 2D culture or 3D culture, Strieder-Barboza et al. proved that adipocytes from different sites of adipose tissues have different metabolic profiling in murine obesity (Strieder-Barboza et al., 2020).

Under the conditions of obesity, excessive FFAs will lead to glucose and lipid metabolism disorders, which eventually weaken the PI3K-AKT signaling pathway, thereby inducing insulin resistance (Bathina and Das, 2018), which will further affect the PI3K-AKT signaling pathway. This ultimately leads to a vicious cycle of obesity and type II diabetes. In addition, the abnormality of the PI3K-AKT signaling pathway is the most common genomic abnormality in breast, ovarian, bladder, and other cancers (Serra et al., 2008; Aziz et al., 2018; Costa et al., 2018; Liu et al., 2018). Rap1 is a small G protein of the Ras superfamily that is present in a variety of important cellular processes, which regulate cell survival and proliferation through the regulation of PI3-AKT. Ras proteins can act as molecular switches that control the integrity of actin cytoskeleton, regulate cell proliferation, cell differentiation, cell adhesion, apoptosis, and cell migration. Overactive Ras signaling can lead to cancer (Goodsell, 1999). The Ras signaling pathway activates the mitogen-activated protein (MAP) kinase cascade and the PI3K-AKT-mTOR pathway, participates in cell growth and division, stimulates protein synthesis, and inhibits cell apoptosis. In conclusion, VAT-derived ASCs may make patients with excessive VAT more susceptible to insulin resistance by activating the PI3K-Akt signaling pathway, thus increasing the risk of multiple tumor diseases in obese patients.

Phagosomes, which are individually involved in EASCs, are associated with inflammation by producing proinflammatory cytokines, such as IL-1β, IL-6, TNF-α, and IL-12 (Aderem, 2003); PI-3 kinase and PLC are also involved in triggering inflammation.

The unique signaling pathways of PASCs were the HIF-1 signaling pathway, p53 signaling pathway, cell cycle, and apoptosis. Previous studies indicated that the HIF-1 signaling pathway is associated with multiple tumor progressions (Zhong et al., 1999; Talks et al., 2000), and dysregulation of cell cycle components may also lead to tumorigenesis, which results in cell proliferation by p53 or other signaling pathways (Champeris Tsaniras et al., 2014). Inhibition of apoptosis can also lead to a variety of cancers, inflammatory diseases, and viral infections (Kaczanowski, 2016). Therefore, the increased risk of cancer in obese or overweight patients may be related to PASCs.

In our combined multi-omics analysis of SASCs and EASCs, the hub gene *Acat2* was found to be associated with nine metabolites in 10 metabolic pathways. *Acat2* is a cellular enzyme that converts cholesterol and fatty acids into cholesterol esters and is mainly expressed in the liver and intestine (Anderson et al., 1998). Studies have shown that atherosclerosis is reduced in mouse atherosclerosis models when *Acat2* is deficient (Willner et al., 2003); *Acat2*-deficient mice are resistant to diet-induced hypercholesterolemia or cholesterol stone formation (Buhman et al., 2000). Lower levels of *Acat2* activity in the human liver can delay the development of atherosclerosis (Parini et al., 2004); therefore, improving the stability of *Acat2* may be an effective treatment strategy for type II diabetes (Wang et al., 2017). Consistent with the above findings, the higher expression of *Acat2* in EASCs rather than SASCs may be more related to fat metabolism and insulin resistance.

In the multi-omics analysis of SASCs and PASCs, three hub genes, *Rrm1*, *Rrm2*, and *Gla*, were mainly involved. In 2002, the International Agency for Research on Cancer (IARC) reported that obese and overweight patients had an increased risk of colon cancer, postmenopausal breast cancer, endometrial cancer, renal cancer, and esophageal cancer (Basen-Engquist and Chang, 2011). In 2003, a cohort study by Calle (Calle et al., 2003) involving 9,000,000 adults in the United States showed that obesity also increases the risk of other cancers, such as liver cancer, pancreatic cancer, non-Hodgkin lymphoma, and myeloma. In early reports, *Rrm1* and *Rrm2* are regulatory subunits of ribonucleotide reductase, negative rate-limiting enzymes for DNA synthesis and repair, playing crucial roles in cell proliferation, invasion, migration, angiogenesis, aging, and tumorigenesis (Nordlund and Reichard, 2006). *Rrm1* and *Rrm2* are widely found in 25 different human tissues, which is normal at low expression levels. However, *Rrm1* and *Rrm2* are overexpressed in various malignant tumors, acting as tumor drivers (Chen et al., 2019). At present, the mechanism by which obesity is associated with the risk of multiple malignant tumors is still unknown. We speculate that the increased risk of some malignant tumors may be associated with the overexpression of *Rrm1* and *Rrm2*, and the main signaling pathways involved may be related to the HIF-1 signaling pathway, p53 signaling pathway, cell cycle, and apoptosis.

*Gla* is widely found in 27 different tissues in the human body, including the adrenal gland, bone marrow, brain, adipose tissue, heart, kidney, liver, lung, lymph node, uterus, placenta, spleen, stomach, and other tissues. *Gla* gene inactivity or deficiency will lead to Fabry disease, mainly affecting the heart, kidney, gastrointestinal tract, pancreas, skin, lung, and nervous system, ultimately leading to multiple organ damage. Patients showed nerve injury in the early stage of Fabry disease, with the main symptoms of paroxysmal burning pain in the extremities in childhood and then relief in adulthood. With the increase of age, the severity of kidney injury increases. Some patients present with renal failure at approximately 30 years old (Germain, 2010) and die from uremia or complications of cardiovascular and cerebrovascular diseases between 40 and 50 years old (Li et al., 2019). The downregulation of *Gla* gene expression in PAT may lead to renal failure and some cardiovascular and cerebrovascular diseases through the renin-angiotensin-aldosterone system and renal parenchymal injury.

## Materials and Methods

### Experimental Animals

Six, 6-week-old male Sprague Dawley rats (Vitronix, China) were purchased from Vital River Biological Co., (Beijing, China). Then the rats were raised under SPF conditions according to the breeding regulations and adapted to the environment for 3 days. All experiments were conducted with the approval of the Animal Experimental Ethics Committee. All procedures were approved (approval number IACUC-1712010) by the Experimental Animal Care and Use Committee of Nanjing Medical University and conducted in accordance with the Guide for the Care and Use of Laboratory Animals (NIH publication No. 85-23, revised 1996).

### Isolation and Culture of Adipose-Derived Mesenchymal Stem Cells

**FIGURE 9:**
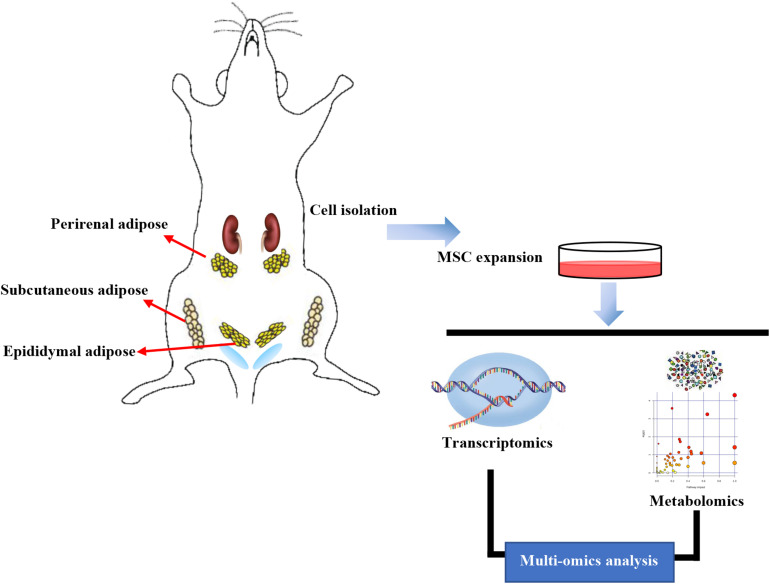
Workflow in our study. Subcutaneous adipose-derived mesenchymal stem cells (SASCs), perirenal adipose-derived mesenchymal stem cells (PASCs), and epididymal adipose-derived mesenchymal stem cells (EASCs) were isolated from Sprague Dawley rats. RNA and metabolites were extracted and sequenced using transcriptomics and metabolomics analyses.

Rats were starved for 12 h before isolating ASCs. First, rats were anesthetized with 2% isoflurane to induce sacrifices, soaked in 75% ethanol for whole-body disinfection, removed from alcohol, and placed on a sterile ultra-clean table. The limbs were fixed, and the SAT was obtained from the groin. Perirenal and EAT was obtained from the kidney and testis, and the tissue was rinsed in precooled PBS. The washed adipose tissue was transferred into the cell platform, muscle and lymph tissue were removed, tissue was cut into 1–2 mm pieces, and collagenase type I was added. After digestion, all the suspensions collected were filtered through a nylon filter (Dutscher, 100 μm), centrifuged at 1,000 rpm for 5 min, and the supernatant was discarded. DMEM/F12 medium containing 10% fetal bovine serum (FBS) was added to the sample. Then the suspension was evenly spread in culture dishes and cultured into a 37^°^C incubator for 2 h. The upper-medium was discarded, high glucose medium containing 10% FBS was added, and SASCs, PASCs, and EASCs were obtained. After culturing at 37^°^C in an incubator for 3 days, RNA and metabolites were extracted from the third passage (P3) cells and sequenced by transcriptomics and metabolomics, respectively (Figure 9).

### RNA Extraction and Transcriptome Sequencing

P3 SASCs, PASCs, and EASCs were cultured in 12-well plates with six replicates in each group. Each well was ground with 1 ml Trizol solution, placed at room temperature for 5 min, and then aspirated. RNA was separated using chloroform (mixture:chloroform = 5:1) and precipitated by isopropanol and absolute ethanol. Finally, RNA was precipitated with DEPC water, and then stored at −80^°^C for subsequent transcriptome sequencing (Guangzhou Ruibo company, Guangzhou, China). In order to ensure the accuracy of sequencing data, the RNA samples were detected by agarose gel electrophoresis, Nanodrop One/One^c^, Qubit 2.0, and Agilent 2100 to estimate the effectiveness, concentration, purity, and integrity of RNA samples, and the cDNA library could be constructed only after the detection results meet the requirements. The HiSeq 3000 System (Illumina^®^) was used for sequencing that generated 150 bp paired-end reads. Before data analysis, the adaptor was removed by cutadapt software (version 3.1), and the low-quality sequence was removed by FastX-Toolkit software (version 0.0.13). The alignment was performed with TopHat softwar (version 2.1.1) (–read-mismatches = 2, –read-gap-length = 2), and the reference genome was rn6 (comparison efficiency >80%). Based on Bowtie v2, transcriptome sequencing reads were compared with the reference genome and splicing junction between exons were identified. Fragments per kilobase of transcript per million reads mapped (FPKM) was used as an index to measure gene expression. The differential expression analysis was performed by DEGseq, which was based on binomial distribution and combined with Fisher’s exact test and likelihood ratio test, and the genes with fold change >2 and FDR <0.05 were identified as DEGs. KOBAS v2.0 R language package was used for functional enrichment analysis. The upregulated or downregulated DEGs were subjected to network analyses^1^ as previously described (Schuierer et al., 2010).

### Metabolite Extraction and Metabolomic Sequencing

P3 SASCs, PASCs, and EASCs were cultured in 10 cm diameter cell culture dishes with six replicates in each group. When the cells grew to 90% their original size, the metabolites were extracted as follows: the cells were washed three times with PBS, then 0.5 ml of 80% (HPLC grade) methanol was added. After repeated blowing and washing of the residual cells, as much of the cell mixture was collected as possible, centrifuged at room temperature at high speed, and the supernatant was dried with nitrogen and stored at −80^°^C for liquid chromatography-mass spectrometry (LC-MS/MS).

Before metabolomic sequencing, the sample was reconstituted with 10 μl of double-distilled water (HPLC grade). Then metabolites were separated by reverse-phase LC (HP1100, Agilent Technologies, Santa Clara, CA, United States) using a reversed-phase XBridge C18 column (1.7 μm particle size, 1 × 150 mm, Waters, Milford, MA, United States) and 2 μl of each sample was injected on the C18 column for analysis with a 0.4-ml/min flow rate. A quality control sample was run first followed by 10 test samples. Mobile phase A consisted of H_2_O/0.1% formic acid and mobile phase B consisted of acetonitrile with 0.1% formic acid which was followed by a program: 1% B at 0–1 min, 15% B at 3 min, 70% B at 5 min, 85% at 9 min, 100% at 10–12 min, and subsequently return to the initial conditions with 2 min for equilibration. Furthermore, the MS program was conducted on a 6545 Quadrupole-Time of Flight system (all devices from Agilent Technologies, Santa Clare, CA, United States) as follows: both positive and negative ion modes with drying gas 300^°^C flow 6 L/min, sheath gas 340^°^C flow 11 L/min, nebulizer gas 35 psig, capillary voltage 4,000 V, and fragmental voltage 135 V. The data collection (MS: 100–3,200 *m*/*z*, MS/MS: 30–3,200 *m*/*z*) was acquired by both centroid and profile stored in autoMSMS scan mode with reference masses at *m*/*z* 112.05087 and 922.009798 were set as online accurate mass calibration.

MassHunter Workstation software (version B.07.00; Agilent Technologies) was used to export mzdata format from the acquired MS data (.d). Data pretreatment procedures were performed by using XCMS Online^2^ for peak discrimination, filtering, and alignment. A table of the intensities of all the peaks was created with the retention time and the mass to ratio for each ion after being aligned by time domain, automatic integration, and extraction of the peak intensities. Then, metabolites were qualitatively analyzed with MS/MS spectra by retrieving and matching the data from Metlin, Massbank, and Human Metabolome Database (HMDB).

The MS peak list was subjected to MetaboAnalyst 3.0 for statistical analysis. PCA and pathway analysis were performed with the web-based software MetaboAnalyst 3.0^3^. Briefly, missing values were replaced by 1/5 of min positive values of their corresponding variables. Sample normalization by sum and data scaling by auto scaling were applied (mean centered and divided by the standard deviation of each variation) (Supplementary Figures 2A,D). Fold change analysis was used to compare the absolute value of change between two groups (Fold change threshold: two). Nonparametric tests were employed according to the normalized data (Adjusted *P*-value (FDR) cutoff <0.05). The different metabolites were subjected to enrichment analysis and pathway analysis in MetaboAnalyst 3.0 by KEGG.

### Integrative Analysis of Multi-Omics Data

Top 100 DEGs and differential metabolites (top 50 upregulated and top 50 downregulated) were selected for further correlation analysis by calculating Pearson’s correlation coefficients on the relative gene expression and metabolite content data between different VASCs with SASCs (Supplementary Table 9). The correlation value and *P*-value were used to assess the relationship between metabolites with genes which are shown by Heatmap and association analysis network diagram.

The DEGs and differential metabolites were subjected to KEGG Mapper^4^ to get the common pathway. KGML file, a subset in the KEGG database, contains the relationships of the graph objects and information about lineal homologous genes in the KEGG genes database. The differences in genes and metabolites in our study were subjected to the database, and the matched network relationships were set as a basic network. Metabolome and transcriptome relationships were visualized and interpreted using Cytoscape (version 3.4.0), and hub genes were calculated by CytoHubba (a Cytoscape plug-in for identifying hub nodes) through MCC (the top 10 nodes ranked by MCC) and degree (degree >6) algorithm.

## Conclusion

In conclusion, similar to SAT, PAT, and EAT, functional differences were found among SASCs, PASCs, and EASCs in our study. The possible metabolic pathways of these functional differences were found using both transcriptomic and metabolomic analyses, and our data provide a theoretical basis for future studies.

## Data Availability Statement

The datasets presented in this study can be found in online repositories. The names of the repository/repositories and accession number(s) can be found below: https://www.ebi.ac.uk/metabolights/, MTBLS2100 and Gene Expression Omnibus, GSE161312.

## Ethics Statement

The animal study was reviewed and approved by All procedures were approved (approval number IACUC-1712010) by the Experimental Animal Care and Use Committee of Nanjing Medical University and conducted in accordance with the Guide for the Care and Use of Laboratory Animals (NIH publication No. 85-23, revised 1996).

## Author Contributions

XK and WS designed the work and prepared the manuscript. CY, JZ, and TW performed the experiments, conducted the data analysis, and wrote the manuscript. KZ, XW, and JS analyzed and interpreted the data. All authors discussed the results and read and approved the final version of the manuscript for publication.

## Conflict of Interest

The authors declare that the research was conducted in the absence of any commercial or financial relationships that could be construed as a potential conflict of interest.

## Footnotes

1 https://portal.genego.com

2 https://xcmsonline.scripps.edu/

3 http://www.metaboanalyst.ca

4 https://www.kegg.jp/kegg/tool/map_pathway3.html

## Supplementary Material

Supplementary Figure 1 **(A)** Top ten Pathway maps analysis (left panel), Gene Ontology (GO) process analysis (middle panel) and Process networks (right panel) analysis sorting as differentially DEGs in EASCs. **(B)** Top ten Pathway maps analysis (left panel), Gene Ontology (GO) process analysis (middle panel), and Process networks (right panel) analysis sorting as differentially DEGs in PASCs. **(C)** Top scored networks analysis of the differentially DEGs in EASCs. **(D)** Top scored networks analysis of the differentially DEGs in PASCs.

Supplementary Figure 2 **(A)** Normalization of metabolomic data of SASCs and EASCs. **(B)** PCA plot for samples of SASCs and EASCs. **(C)** *T*-tests between SASCs and EASCs. **(D)** Normalization of metabolomic data of SASCs and PASCs. **(E)** PCA plot for samples of SASCs and PASCs. **(F)** *T*-tests between SASCs and PASCs.

Supplementary Figure 3 Hub genes were calculated by CytoHubba through MCC.

Supplementary Figure 4 **(A)** The enriched KEGG pathways of DEGs between SASCs and EASCs. **(B)** The enriched KEGG pathways of DEGs between SASCs and PASCs.

Supplementary Figure 5 Bioinformatics analysis workflow.
