# Supplementary material for: Multi-Omics Analysis to Examine Gene Expression and Metabolites From Multisite Adipose-Derived Mesenchymal Stem Cells
Source: Front Genet. 2021 Feb 18;12:627347. doi: 10.3389/fgene.2021.627347 (PMC7930907; doi:10.3389/fgene.2021.627347)
Supplement: Supplementary Figure 1 — (A) Top ten Pathway maps analysis (left panel), Gene Ontology (GO) process analysis (middle panel) and Process networks (right panel) analysis sorting as differentially DEGs in EASCs. (B) Top ten Pathway maps analysis (left panel), Gene Ontology (GO) process analysis (middle panel), and Process networks (right panel) analysis sorting as differentially DEGs in PASCs. (C) Top scored networks analysis of the differentially DEGs in EASCs. (D) Top scored networks analysis of the differentially DEGs in PASCs. [file Image_1.PDF]

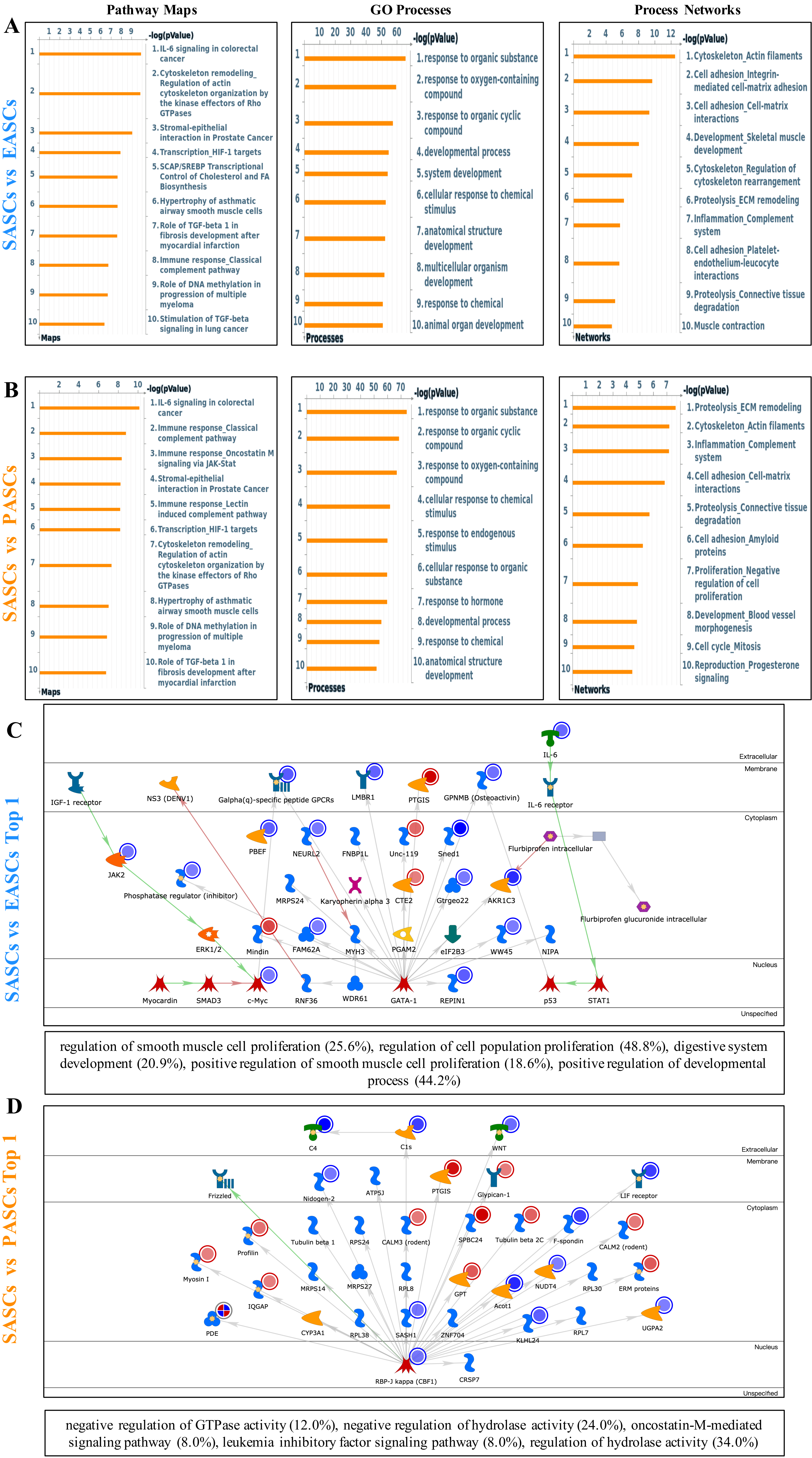

Pathway Maps
GO Processes
Process Networks
A
SASCs vs EASCs
-log(pValue)
1. IL-6 signaling in colorectal cancer
2. Cytoskeleton remodeling_ Regulation of actin cytoskeleton organization by the kinase effectors of Rho GTPases
3. Stromal-epithelial interaction in Prostate Cancer
4. Transcription_HIF-1 targets
5. SCAP/SREBP Transcriptional Control of Cholesterol and FA Biosynthesis
6. Hypertrophy of asthmatic airway smooth muscle cells
7. Role of TGF-beta 1 in fibrosis development after myocardial infarction
8. Immune response_Classical complement pathway
9. Role of DNA methylation in progression of multiple myeloma
10. Stimulation of TGF-beta signaling in lung cancer
Maps
1. response to organic substance
2. response to oxygen-containing compound
3. response to organic cyclic compound
4. developmental process
5. system development
6. cellular response to chemical stimulus
7. anatomical structure development
8. multicellular organism development
9. response to chemical
10. animal organ development
Processes
1. Cytoskeleton_Actin filaments
2. Cell adhesion_Integrin-mediated cell-matrix adhesion
3. Cell adhesion_Cell-matrix interactions
4. Development_Skeletal muscle development
5. Cytoskeleton_Regulation of cytoskeleton rearrangement
6. Proteolysis_ECM remodeling
7. Inflammation_Complement system
8. Cell adhesion_Platelet-endothelium-leucocyte interactions
9. Proteolysis_Connective tissue degradation
10. Muscle contraction
Networks
B
SASCs vs PASCs
1. IL-6 signaling in colorectal cancer
2. Immune response_Classical complement pathway
3. Immune response_Oncostatin M signaling via JAK-Stat
4. Stromal-epithelial interaction in Prostate Cancer
5. Immune response_Lectin induced complement pathway
6. Transcription_HIF-1 targets
7. Cytoskeleton remodeling_ Regulation of actin cytoskeleton organization by the kinase effectors of Rho GTPases
8. Hypertrophy of asthmatic airway smooth muscle cells
9. Role of DNA methylation in progression of multiple myeloma
10. Role of TGF-beta 1 in fibrosis development after myocardial infarction
1. response to organic substance
2. response to organic cyclic compound
3. response to oxygen-containing compound
4. cellular response to chemical stimulus
5. response to endogenous stimulus
6. cellular response to organic substance
7. response to hormone
8. developmental process
9. response to chemical
10. anatomical structure development
1. Proteolysis_ECM remodeling
2. Cytoskeleton_Actin filaments
3. Inflammation_Complement system
4. Cell adhesion_Cell-matrix interactions
5. Proteolysis_Connective tissue degradation
6. Cell adhesion_Amyloid proteins
7. Proliferation_Negative regulation of cell proliferation
8. Development_Blood vessel morphogenesis
9. Cell cycle_Mitosis
10. Reproduction_Progesterone signaling
C
SASCs vs EASCs Top 1
Extracellular
Membrane
Cytoplasm
Nucleus
Unspecified
IL-6
IGF-1 receptor
NS3 (DENV1)
Galpha(q)-specific peptide GPCRs
LMBR1
PTGIS
GPNMB (Osteoactivin)
IL-6 receptor
PBEF
NEURL2
FNBP1L
Unc-119
Sned1
Flurbiprofen intracellular
JAK2
Phosphatase regulator (inhibitor)
MRPS24
Karyopherin alpha 3
CTE2
Gtrgeo22
AKR1C3
Flurbiprofen glucuronide intracellular
ERK1/2
Mindin
FAM62A
MYH3
PGAM2
eIF2B3
WW45
NIPA
Myocardin
SMAD3
c-Myc
RNF36
WDR61
GATA-1
REPIN1
p53
STAT1
regulation of smooth muscle cell proliferation (25.6%), regulation of cell population proliferation (48.8%), digestive system development (20.9%), positive regulation of smooth muscle cell proliferation (18.6%), positive regulation of developmental process (44.2%)
D
SASCs vs PASCs Top 1
C4
C1s
WNT
Frizzled
Nidogen-2
ATP5J
PTGIS
Glypican-1
LIF receptor
Tubulin beta 1
RPS24
CALM3 (rodent)
SPBC24
Tubulin beta 2C
F-spondin
CALM2 (rodent)
Profilin
Myosin I
MRPS14
MRPS27
RPL8
GPT
NUDT4
RPL30
ERM proteins
IQGAP
Acot1
PDE
CYP3A1
RPL38
SASH1
ZNF704
KLHL24
RPL7
UGPA2
RBP-J kappa (CBF1)
CRSP7
negative regulation of GTPase activity (12.0%), negative regulation of hydrolase activity (24.0%), oncostatin-M-mediated signaling pathway (8.0%), leukemia inhibitory factor signaling pathway (8.0%), regulation of hydrolase activity (34.0%)
