# Supplementary figures and images for: Multi-Omics Analysis to Examine Gene Expression and Metabolites From Multisite Adipose-Derived Mesenchymal Stem Cells
Source: Front Genet. 2021 Feb 18;12:627347. doi: 10.3389/fgene.2021.627347 (PMC7930907; doi:10.3389/fgene.2021.627347)

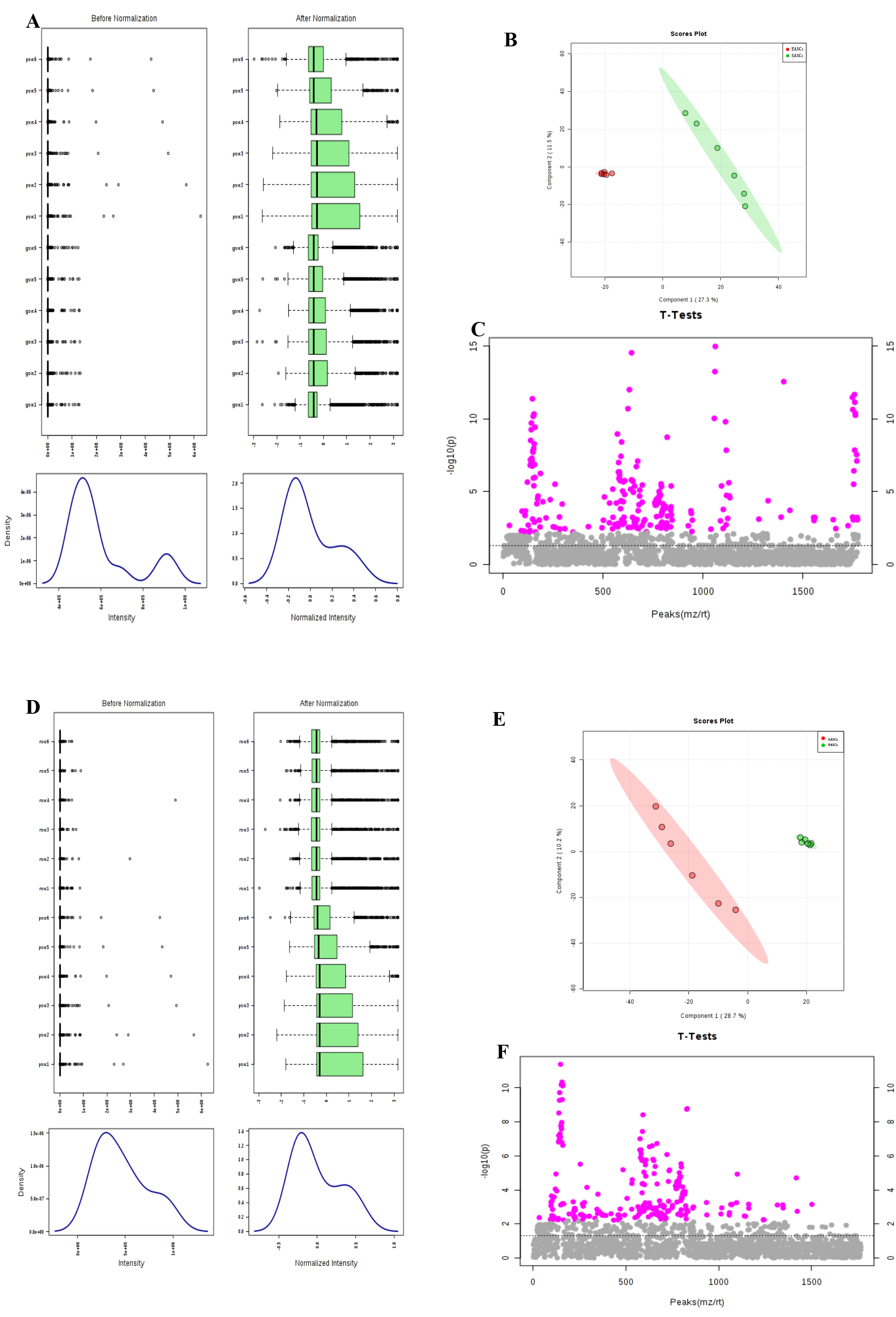

Supplement: Supplementary Figure 2 — (A) Normalization of metabolomic data of SASCs and EASCs. (B) PCA plot for samples of SASCs and EASCs. (C) T-tests between SASCs and EASCs. (D) Normalization of metabolomic data of SASCs and PASCs. (E) PCA plot for samples of SASCs and PASCs. (F) T-tests between SASCs and PASCs. [file Image_2.TIF]

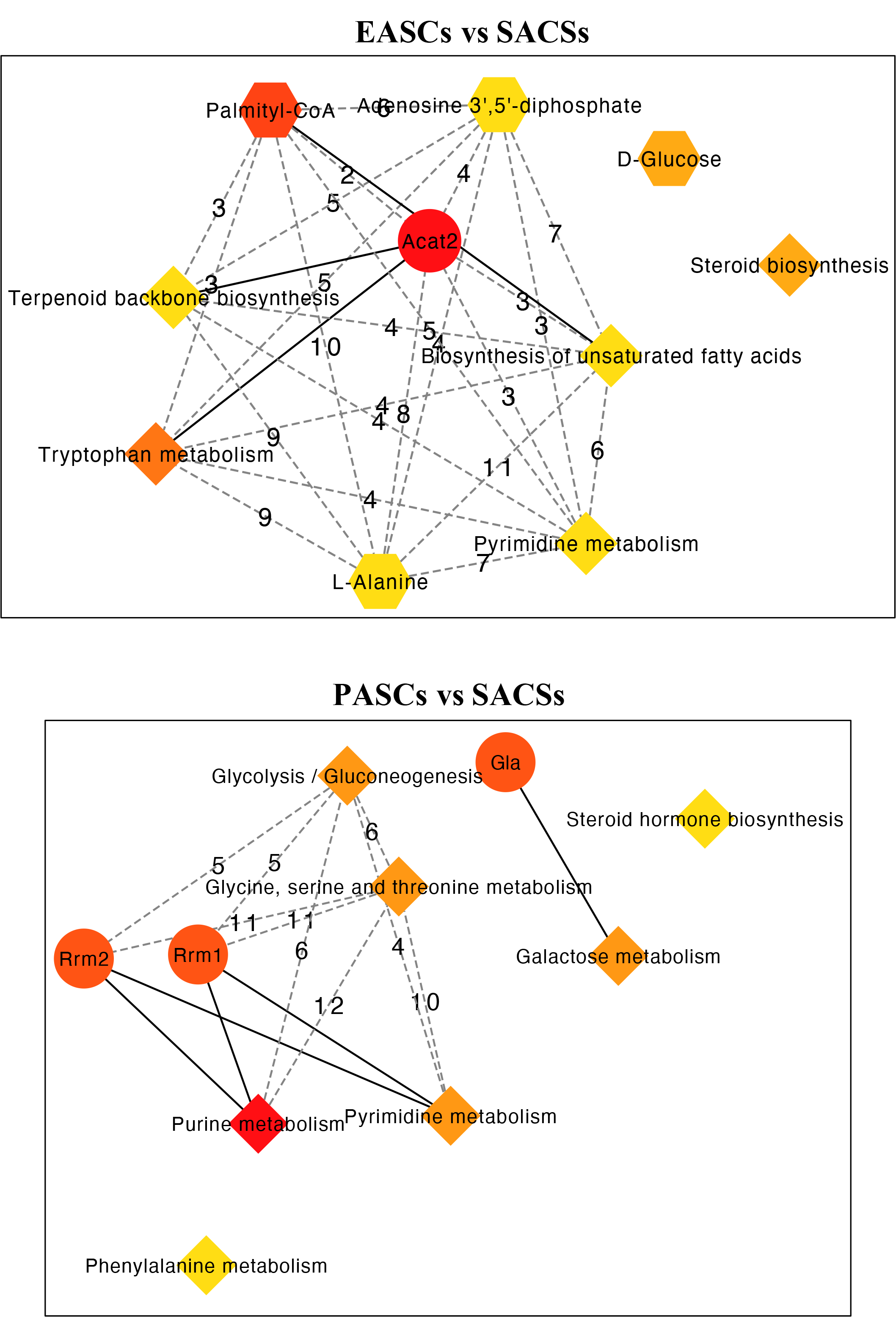

Supplement: Supplementary Figure 3 — Hub genes were calculated by CytoHubba through MCC. [file Image_3.TIF]

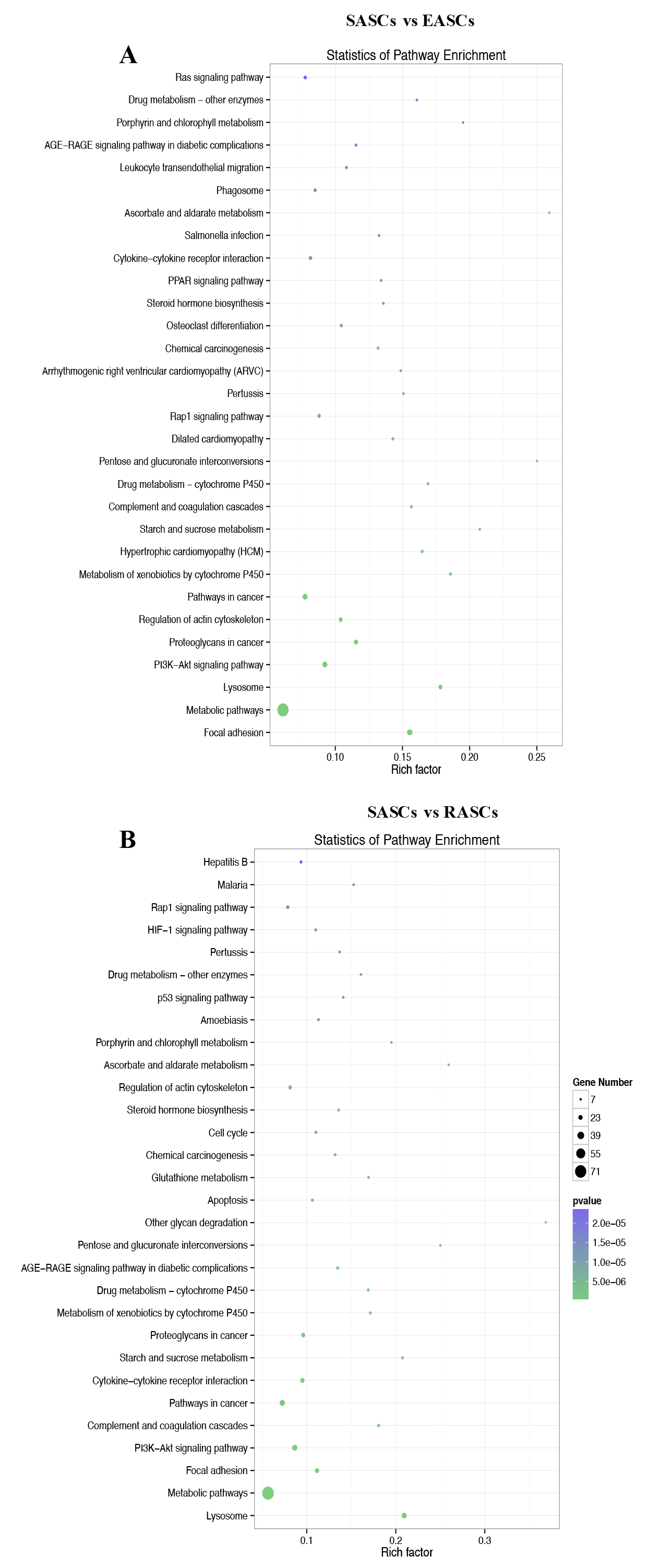

Supplement: Supplementary Figure 4 — (A) The enriched KEGG pathways of DEGs between SASCs and EASCs. (B) The enriched KEGG pathways of DEGs between SASCs and PASCs. [file Image_4.TIF]

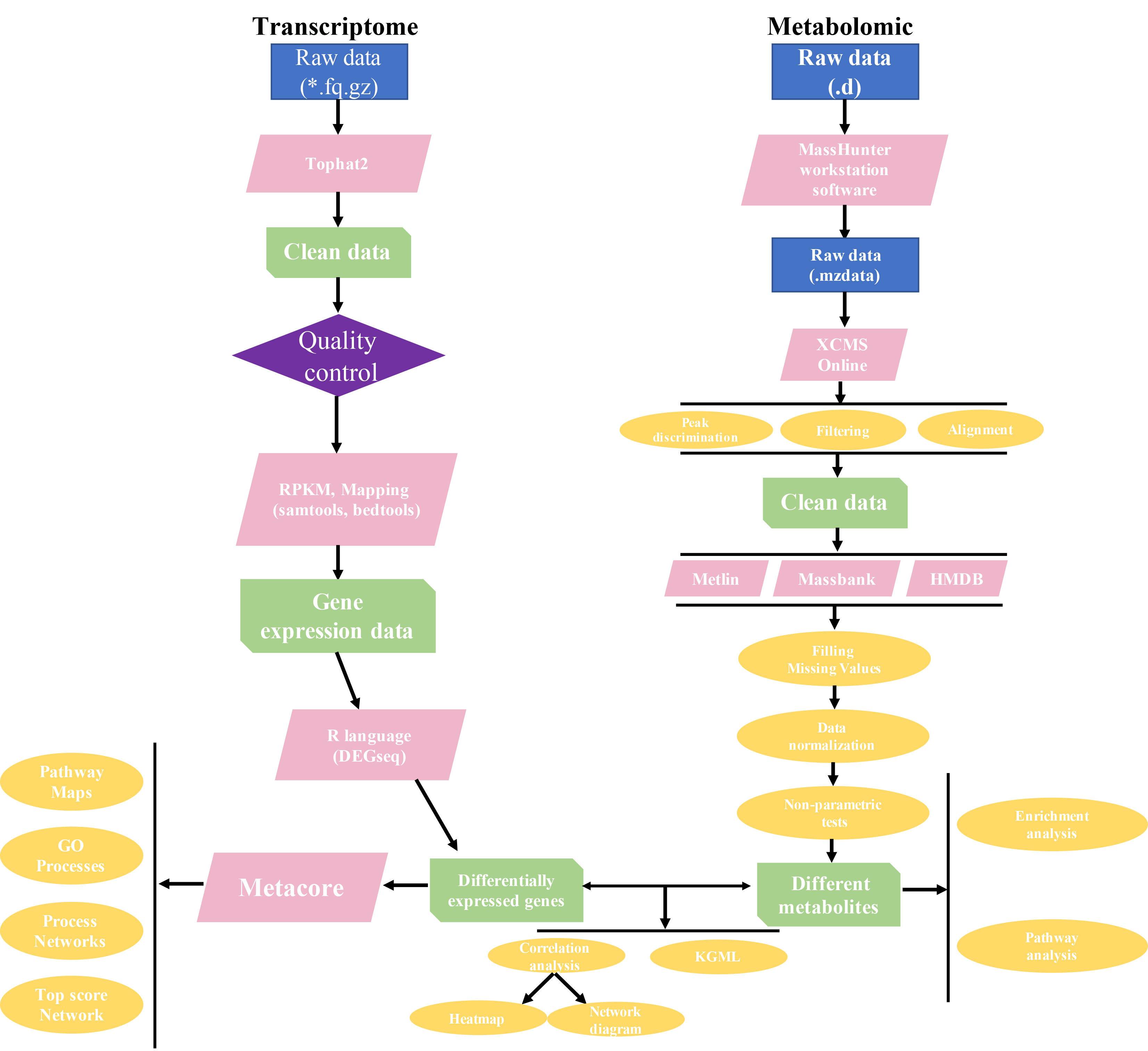

Supplement: Supplementary Figure 5 — Bioinformatics analysis workflow. [file Image_5.TIF]
